# Plasma metabolomic analysis reveals the therapeutic effects of Jiashen tablets on heart failure

**DOI:** 10.3389/fcvm.2022.1047322

**Published:** 2022-12-06

**Authors:** Xinglong Miao, Jiaping Chen, Yangyan Su, Jiayi Luo, Yi He, Jiang Ma, Xin He

**Affiliations:** ^1^School of Chinese Materia Medica, Tianjin University of Traditional Chinese Medicine, Tianjin, China; ^2^School of Chinese Materia Medica, Guangdong Pharmaceutical University, Guangzhou, China; ^3^State Key Laboratory of Core Technology in Innovative Chinese Medicine, Tasly Pharmaceutical Group Co., Ltd., Tianjin, China

**Keywords:** heart failure, Jiashen tablets, LAD ligation, cardiac function, plasma metabolomics

## Abstract

**Background:**

Heart failure is a chronic progressive condition that significantly affects the quality of life of patients with high hospitalization and mortality rates. Jiashen tablets (JST), a Chinese herbal formula, have been reported to be an effective treatment against heart failure, however the underlying mechanisms remain obscure. This study was designed to determine the effect of JST on the treatment of heart failure and delineate the underlying mechanisms by an untargeted metabolomics approach.

**Materials and methods:**

The chronic heart failure model was established by the permanent ligation of the left anterior descending coronary artery in rats. The cardiac functions of rats, including left ventricular ejection fraction (LVEF) and fractional shortening (LVFS), left ventricular internal diameter end diastole (LVIDd) and end systole (LVIDs), and interventricular septum thickness in diastole (IVSd) and in systole (IVSs), were measured by echocardiography. Biochemical analysis and histopathological examination were also performed to evaluate therapeutic effects of JST for treating heart failure. UHPLC-QTOF-MS/MS coupled with multivariate statistical analyses were applied for plasma metabolic profiling to identify biomarkers and potential mechanisms of JST in the treatment of heart failure.

**Results:**

Jiashen tablets could improve the cardiac function of heart failure rats and thus ameliorate heart failure via enhancing rat LVEF and LVFS and decreasing LVIDd, LVIDs, IVSd, and IVSs. Results of biochemical analysis and histopathological examination revealed that JST could reduce the serum lactate dehydrogenase (LDH) activity and the level of NT-pro BNP, markers of heart failure and myocardial damage, and inhibit myocardial fibrosis. Furthermore, in metabolomics analysis, a total of 210 metabolites with significant differences were identified between heart failure rats and normal rats, among which 29 metabolites were significantly restored after JST treatment. These metabolites were primarily involved in tryptophan metabolism, branched-chain amino acid metabolism, fatty acids β-oxidation, and glycerophospholipid metabolism.

**Conclusion:**

The present study illustrated the therapeutic effect of JST for the treatment of heart failure and delineated the underlying mechanisms mainly relating to the regulation of amino acid metabolism and lipid metabolism in heart failure rats.

## Introduction

Heart failure is a chronic progressive cardiovascular disease characterized by abnormal cardiac function, myocardial hypertrophy, and myocardial remodeling due to impaired systolic or diastolic function of the heart (1, 2). The prevalence of heart failure in 2021 is estimated to be 1.5–1.9% in the United States and 1–2% in Europe (3). Epidemiological studies showed that the incidence of heart failure was almost flat or decreasing; however, the burden of mortality and hospitalization remained unabated despite significant efforts putting in the treatment and management of heart failure (3). Oxidative stress and inflammatory responses, excessive myocardial fibrosis, abnormalities in calcium cycling, and abnormal mitochondrial function represent the key mechanisms underlying heart failure (4). Drugs currently used to treat heart failure mainly include angiotensin-converting enzyme inhibitors, β-blockers, salt corticosteroid receptor antagonists, aldosterone receptor antagonists, and diuretics (5). However, the long-term use of those drugs can lead to adverse effects such as hypotension, fluid depletion and electrolyte disturbances (6, 7). Therefore, there remains a large unmet need for new therapies in the treatment of heart failure.

Herbal compound formulas of traditional Chinese medicine (TCM) are a specific combination of multiple herbs, which have the advantage of fewer side effects for treating multi-factorial diseases. Several herbal compound formulas of TCM have been clinically used for the treatment of heart failure, such as Buyanghuanwu decoction, Qishenyiqi dropping pills, and Qiliqiangxin (QLQX) capsules (8–10). Jiashen tablets (JST), which consists of Huangqi (Astragali Radix), Danshen (Salviae Miltiorrhizae Radix et Rhizoma), Xiangjiapi (Periplocae Cortex), Sanqi (Notoginseng Radix et Rhizoma), Yimucao (Leonuri Herba), Chenpi (Citri Reticulatae Pericarpium), Guizhi (Cinnamomi Ramulus), and Tinglizi (Descurainiae Semen Lepidii Semen), is a clinical prescription used for treating heart failure. Previous chemical studies showed that there were 68 components identified in JST, mainly including phenolic acids, tanshinones, flavonoids, and their glycosides, cardiac glycosides, triterpene saponins, and C21 steroids (11). It has been reported that JST could enhance cardiac function and alleviate heart failure through inhibiting myocardial inflammation and apoptosis, intervening in the neuroendocrine system, and preventing adverse ventricular remodeling after acute myocardial injury (12–16). However, the mechanisms of JST in the treatment of heart failure have not been well-characterized, which has become an obstacle to its clinical application.

Metabolomics is a top-down, high-throughput analytical tool centered on the detection, analysis and identification of metabolites (17), which can be used to identify the dynamic processes of endogenous biomarkers, discover early disease markers of drugs, and explore the mechanisms of drugs on diseases (18). Therefore, in order to explore possible mechanisms of JST for treating heart failure, an untargeted metabolomics based on UHPLC-QTOF-MS/MS analysis was performed in the present study. The rat model of heart failure was established by the ligation of left anterior descending coronary artery (LAD) and monitored by measuring cardiac functions and biochemical and histopathological changes. Potential biomarkers modulated by JST in heart failure rats were identified by various multivariate statistical analyses and the mechanisms of JST for treating heart failure were proposed.

## Materials and methods

### Reagents

Jiashen tablets was supplied by Tasly Pharmaceutical Group Co., Ltd. (Tianjin, China). Captopril was purchased from Shanghai Pharmaceutical Group Corp. (Shanghai, China). QLQX capsules were purchased from Shijiazhuang Yilin Pharmaceutical Co. (Hebei, China). NT-pro BNP Elisa Kit was obtained from Cloud-Clone Corp. (Wuhan, China, batch number: CEA485Ra). LDH Kit and Masson’s Trichrome Stain Kit were purchased from Nanjing Jiancheng Bioengineering Inc. (Nanjing, China). Hematoxylin and Eosin (H&E) Staining Kit was purchased from Biosharp Inc. (Beijing, China). HPLC-grade methanol and acetonitrile were obtained from Merck (Darmstadt, Germany). Formic acid and 2-Chlorophenylalanine were bought from Thermo Fisher Scientific (MA, US).

The reference standards, including 4-methoxy-salicylic acid, rosmarinic acid, ononin, notoginsenoside R1, periplogenin, ginsenoside Re, ginsenoside Rg1, formononetin, periplocymarin, ginsenoside Rb1, astragaloside IV, periplocoside M, ginsenoside Rd were purchased from Chengdu Must Bio-technology Co. LTD (Chengdu, China). The total ion chromatograms for mass spectrometric analysis of JST and those mixed reference standards are shown in Supplementary Figure 1, and 12 typical constituents were identified in JST by comparing the retention time and mass spectrum with those reference standards. The content of these identified constituents in JST was determined by the single-point external standard method and was listed in Supplementary Table 1.

### Animals and experimental design

Animal study protocols were approved by the Committee on Laboratory Animal Care and Use of Guangdong Pharmaceutical University (Guangzhou, China), in accordance with the National Institutes of Health guide for the care and use of laboratory animals. A total of 52 Sprague-Dawley rats (180–220 g) were obtained from Guangdong Medical Laboratory Animal Center [license NO. SCXK (yue) 2018-0002]. The animals were housed at 20–25°C, 40–70% humidity and 12 h dark/light cycle conditions with free access to a standard chow diet, and tap water *ad libitum*. All rats were exposed to an “adaptive feeding” paradigm for a week before the start of experiments.

**FIGURE 1:**
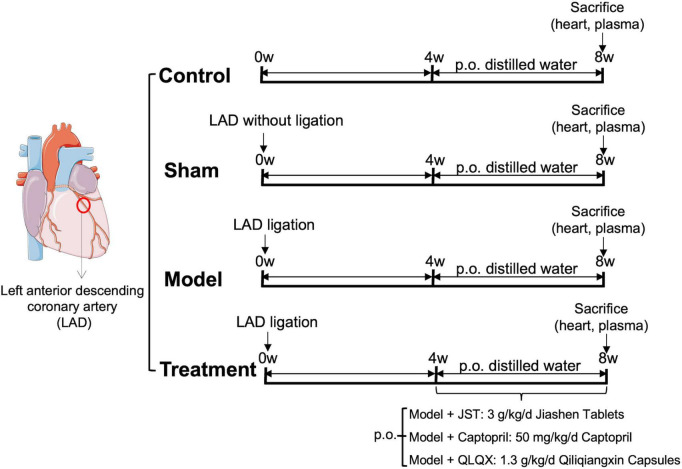
Left anterior descending coronary artery (LAD) ligation for the establishment of rat heart failure model and dosage regimens.

The permanent ligation of the left anterior descending coronary artery (LAD) in 40 rats was performed to establish a heart failure model. Briefly, rats under anesthesia (pentobarbital 50 mg/kg, Deshang Pinshan Biological Technology, Chengdu, China) were placed in a supine position and intubated. Then, rats underwent surgical opening of the chest and were ventilated with an automatic breathing apparatus (tidal volume: 10–12 ml; respiratory rate: 90 cycles/min; respiratory ratio: 1:2). LAD, located between the pulmonary artery and the left auricle, was ligated with 7–0 polypropylene sutures. Four weeks after the surgery, the typical cardiac function index, left ventricular ejection fraction (LVEF), of the rats were measured by echocardiography. Rats with LVEF <60% were considered as heart failure (19, 20). Among those 40 rats, 8 rats died during the operation and 8 rats did not meet the criteria of chronic heart failure after the operation. The remaining 24 rats were divided into four groups (*n* = 6/group), including Model group, JST group, QLQX group, and Captopril group. Rats in sham group underwent the same surgical procedures without the ligation and control rats did not receive any surgeries (*n* = 6/group). Two positive drugs were used in the present study, including a synthetic drug (captopril) and an herbal compound formula (QLQX capsules) (*n* = 6/group). JST, captopril, and QLQX capsules were orally administered to the rats at 3 g/kg/day, 50 mg/kg/day, and 1.3 g/kg/day, which was selected based on their human equivalent dose used in clinical practice. The dosage of JST (3 g/kg) referred to herein is the raw herbs contained, and 1 g of JST is equivalent to 9.5 g of the total amount of eight herbs contained in JST. At 4 weeks post dosing, rats were sacrificed and blood and heart samples were collected for further biochemical, histopathological and metabolomic analyses. Prior to the sacrifice, the cardiac function of the rats was monitored by echocardiography. The specific dosage regimens are shown in Figure 1.

### Measurement of cardiac function

Rats in individual groups were subjected to echocardiography using VisualSonics Vevo 2100 Imaging System (VisualSonics, Canada). The left parasternal long-axis view for M-mode imaging was recorded. The following indexes were measured, including LVEF, left ventricular fractional shortening (LVFS), left ventricular internal diameter end diastole (LVIDd) and end systole (LVIDs), and interventricular septum thickness in diastole (IVSd) and in systole (IVSs).

### Biochemical analysis and histopathological examination

The collected heart tissues were fixed in 4% paraformaldehyde for 24 h and embedded in paraffin blocks, which were further sectioned at 5 μm and stained with H&E and Masson’s trichrome. The stained slices were observed under color digital camera (DP72, Olympus, Japanese). The fibrosis fraction was analyzed by Image-Pro Plus software in the infarcted border zone.

The level of lactate dehydrogenase (LDH) activity in rat serum was measured using the assay kits obtained from Jiancheng (Jiancheng Biotech Co., Ltd., Nanjing, China) by automatic chemistry analyzer (ELX800, BioTek, US). The concentration of NT-pro BNP in the serum was detected by an enzyme-linked immunosorbent assay kit according to the manufactory’s protocols (Cloud-Clone Corp., US).

### Plasma sample preparation

The harvested blood samples were centrifuged at 4,000 rpm for 30 min at 4°C to give the plasma samples. An aliquot of plasma samples (100 μL) was mixed with 300 μL of methanol containing 1 ppm of 2-chlorophenyl alanine, vortexed for 2 min, and incubated at −20°C for 0.5 h. The mixture was then centrifuged at 12,000 rpm for 10 min at 4°C, and the supernatant was subjected to LC-MS/MS system for analysis.

### UHPLC-QTOF-MS/MS analysis

The analysis was conducted on an Agilent 6545 quadrupole time of flight (QTOF) mass spectrometer connecting with an Agilent 1290 ultra performance liquid chromatography (UHPLC) (Agilent Technologies Inc., USA). Chromatographic separation was achieved on ACQUITY UPLC HSS T3 C18 column (1.8 μm × 2.1 mm × 100 mm) at 40°C with a mobile phase of 0.1% formic acid (A) and acetonitrile (B). The elution gradient was as follows: 0–11 min, 95% A; 11–12 min, 10% A; 12–12.1 min, 10% A; 12.2–14 min, 95% A. The flow rate was 0.40 mL/min and the injection volume was 2 μL.

The mass spectrometry was performed under both positive and negative ion modes. The heated electrospray ionization parameters are as follows: sheath flow, 11 L/min; gas flow, 8 L/min; spray voltage, 250 V for positive ionization and negative ionization; fragment voltage, 135 V; gas temperature, 325°C; sheath temperature, 325°C; nebulizer pressure, 40 psi.

### Data processing and statistical analysis

The original data obtained from UHPLC-MS/MS analysis were converted into mzML format by MSConvert^1^ (provided by ProteoWizard). The XCMS program^2^ was used for peak extraction, alignment, and retention time correction. The resultant data was imported to R statistical scripting language (version 3.6.1) for multivariate statistical analyses, including principal component analysis (PCA), partial least squares-discriminant analysis (PLS-DA), and orthogonal PLS-DA analysis (OPLS-DA). In the OPLS-DA analysis, metabolites with variable importance in the projection VIP ≥ 1, *P* < 0.05, and fold change ≥ 2 or fold change ≤ 0.5 were selected as potential biomarkers. The biomarker identification was achieved by searching MassBank,^3^ HMDB,^4^ and METLIN.^5^

All data are expressed as mean ± SD. Statistical analysis was performed on Graphpad Prism 9.0. Student’s *t*-test was used for comparison between two groups and One-way ANOVA (analysis of variance) with Dunnett’s multiple comparison post-test was used for comparison among three or more groups. A *P* < 0.05 was considered statistically significant.

## Results

### Effects of Jiashen tablets on cardiac function of heart failure rats

**FIGURE 2:**
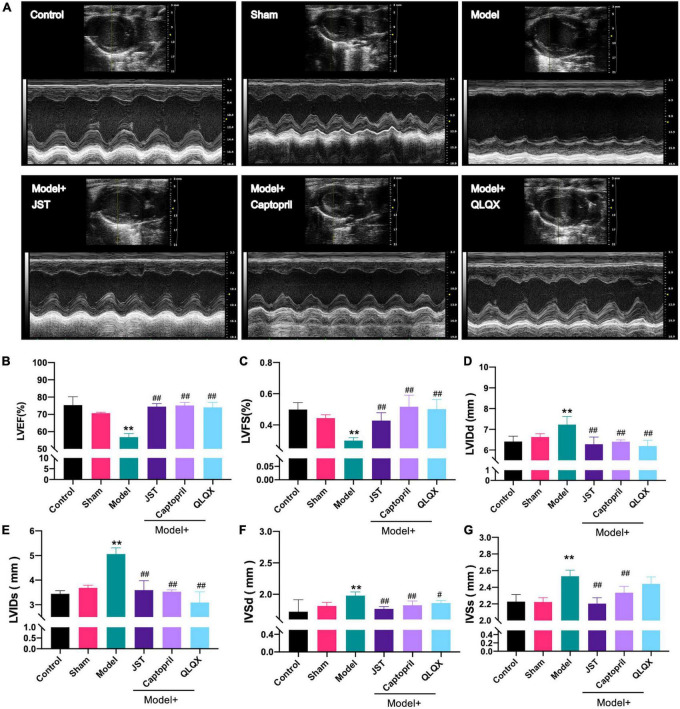
Effects of JST on cardiac function of heart failure rats. Representative M-mode echocardiography images from experimental rats **(A)** and the statistical echocardiographic values, including LVEF **(B)**, LVFS **(C)**, LVIDd **(D)**, LVIDs **(E)**, IVSDd **(F)**, and IVSDs **(G)**. All data were expressed as mean ± SD (*n* = 4–6). **P < 0.01, vs. Sham group; ^#^*P* < 0.05, ^##^*P* < 0.01, vs. Model group.

To investigate the therapeutic effect of JST on heart failure, the cardiac function of rats in individual groups was examined using the small animal ultrasound imaging system. As shown in Figure 2, no significant difference was observed between Sham and Control groups with respect to the tested cardiac ultrasound parameters, including LVEF, LVFS, LVIDs, LVIDd, IVSd, and IVSs. Rats in Model group showed remarkably declined LVEF and LVFS (Figures 2B,C) and significantly increased LVIDd, LVIDs, IVSd, and IVSs (Figures 2D–G) compared to that in Sham group, which indicated that the heart failure model was successfully established. Compared to Model group, treatment of JST, captopril, and QLQX significantly enhanced LVEF and LVFS (Figures 2B,C) and reduced LVIDd, LVIDs, IVSd, and IVSs (Figures 2D–G), suggesting that these three agents can improve the cardiac function of heart failure rats caused by LAD ligation. Moreover, there was no significant difference of the tested parameters among the three treatment groups (Figures 2B–G), which indicated a similar cardioprotective effect of JST, captopril, and QLQX.

### Effects of Jiashen tablets on serum cardiac enzymes and histopathological changes in heart failure rats

**FIGURE 3:**
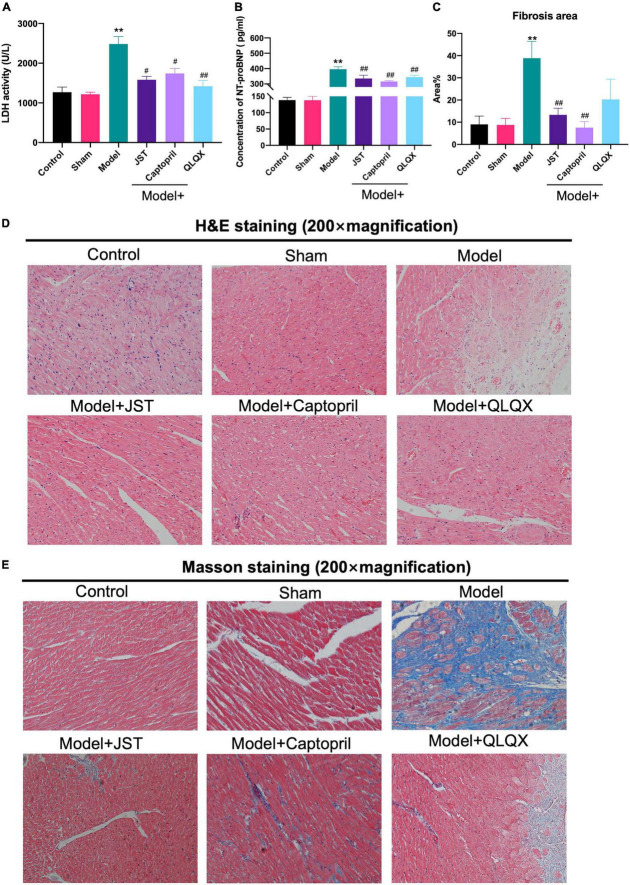
Effects of JST on serum cardiac enzymes and histopathological changes in heart failure rats. Serum LDH activity **(A)**. Serum concentration of NT-pro BNP **(B)**. Quantitative assessment of fibrosis area (%) in Masson’s trichrome-stained heart sections **(C)**. Representative heart sections stained with H&E **(D)** and Masson’s trichrome **(E)**. All data were expressed as mean ± SD (*n* = 4–6). **P < 0.01, vs. Sham group; ^#^*P* < 0.05, ^##^*P* < 0.01, vs. Model group.

Lactate dehydrogenase is a marker of myocardial injury (21) and NT-pro BNP is known as a diagnostic and prognostic biomarker for heart failure (22). To further validate the therapeutic effect of JST on heart failure, serum LDH activity, and NT-pro BNP levels were measured. The results showed that LDH activity (Figure 3A) and NT-pro BNP levels (Figure 3B) significantly elevated in Model rats compared to that in Sham group. JST, captopril, and QLQX significantly reduced the increase of LDH activity and NT-pro BNP levels in Model group and no significant difference was observed among the three treatment groups (Figures 3A,B).

H&E staining and Masson staining were performed to investigate the morphological changes and myocardial fibrosis of the rat heart. As illustrated in Figures 3D,E, compared to Sham group, Model group showed massive swelling of cardiomyocytes with vacuolated lesions and marked myocardial interstitial fibrosis. In addition, the infiltration of inflammatory cells was occasionally observed in Model group. Myocardial fibrosis and inflammatory cell infiltration were significantly reduced in Model + JST, Model + Captopril, and Model + QLQX groups, compared to model group (Figures 3D,E). As shown in Figure 3C, the myocardial fibrosis area significantly increased in Model group, but the increase was remarkably reversed by the treatment of JST, Captopril, and QLQX.

### Metabolomics method validation

**FIGURE 4:**
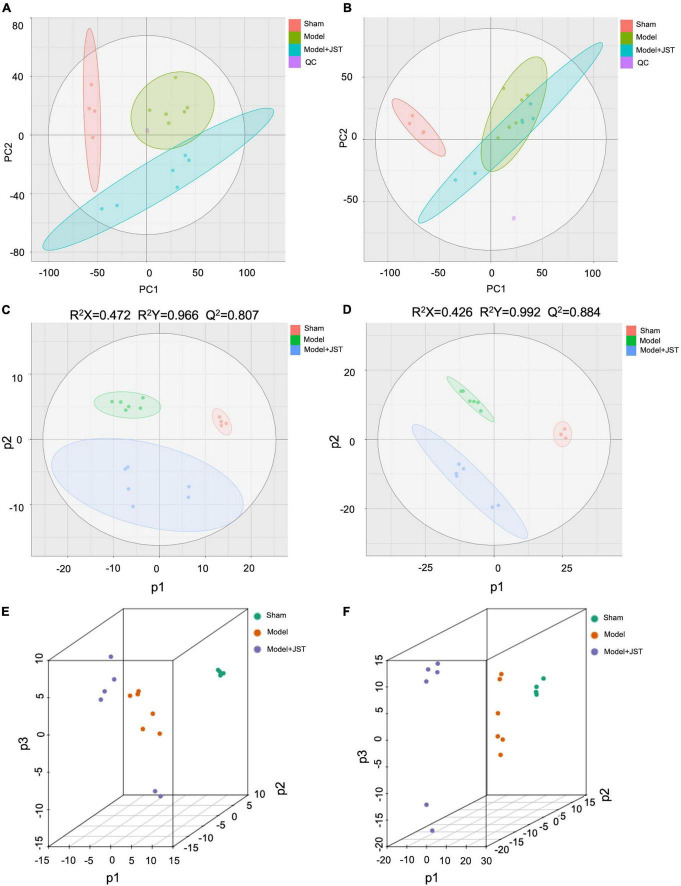
Multivariate statistical analyses. Negative PCA score plots with QC **(A)**; positive PCA score plots with QC **(B)**; negative PLS-DA score plots **(C)**; positive PLS-DA spots **(D)**; negative PLS-DA (3D) spots **(E)**; positive PLS-DA (3D) spots **(F)**.

In the present study, QC samples were generated via pooling 10 μL of plasma samples from each group. PCA results showed that QC samples clustered together (Figures 4A,B), which indicated that the LC-MS/MS system exhibited high stability and reproducibility. Moreover, the overlapped total ion chromatograms (TICs) of QC samples further indicated satisfied reproducibility of the large-scale sample analysis (Supplementary Figure 2). Taken together, the stability and reproducibility of the present metabolomics approach were satisfactory for metabolomics analysis.

### Metabolomics analysis

To further investigate the mechanisms of JST in the treatment of heart failure, UHPLC-Q-TOF-MS/MS was used to analyze the metabolic profiles and metabolites among Sham, Model, and JST groups. A total of 9,203 ions in the positive mode and 6,601 ions in the negative mode were obtained in plasma samples. PCA, an unsupervised pattern recognition method, was firstly used to pattern the plasma metabolic profiles of Sham, Model, and JST groups. As shown in Figure 4A, under negative ion mode, the plasma metabolic profiles of Sham, Model, and JST groups were clearly separated. While under positive ion mode, the Model and JST groups also had a separation trend (Figure 4B).

PLS-DA was further carried out to maximally analyze the difference among the three groups. As shown in Figures 4C,D, PLS-DA score plots showed a clear separation among Sham, Model, and JST groups under both positive and negative ion modes, which was consistent with the PCA results. The evaluation parameters of the PLS-DA model were R^2^X = 0.472, R^2^Y = 0.966, *Q*^2^ = 0.807 (negative ion mode), and R^2^X = 0.426, R^2^Y = 0.992, *Q*^2^ = 0.884 (positive ion mode), indicating that the model possessed goodness of fit and predictive ability. Moreover, 3D PLS-DA plots (Figures 4E,F) also illustrated a shift of JST group to Sham group, which was consistent with the biochemical analysis and pathological examination. These results suggested the metabolic profiles were ameliorated in the plasma of heart failure rats after JST treatment.

### Screen and identification of potential biomarkers

**FIGURE 5:**
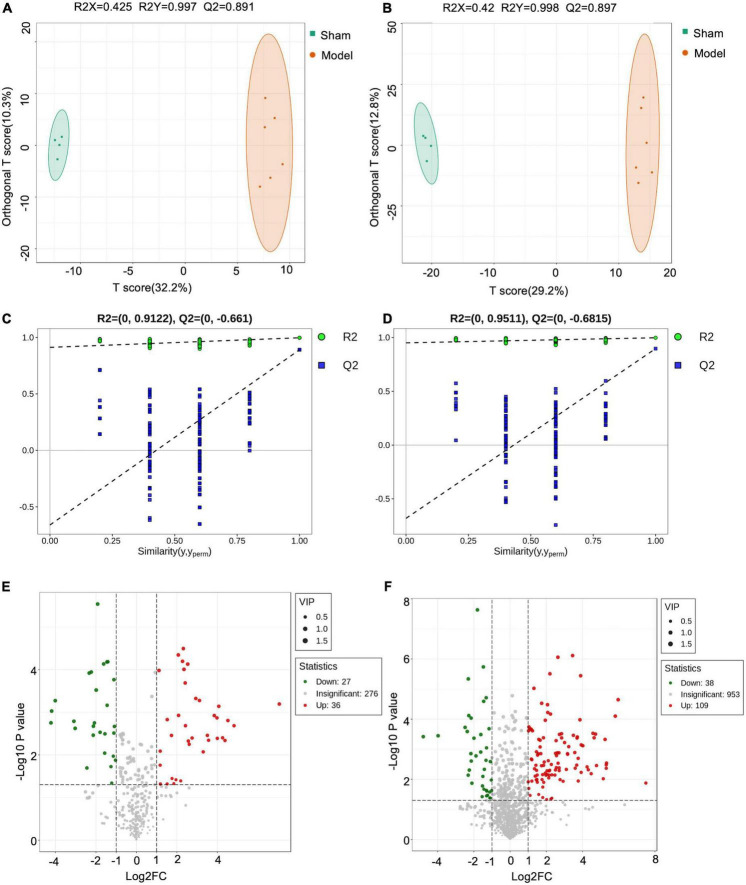
Orthogonal PLS-DA analysis (OPLS-DA) analyses. Negative OPLS-DA score plots **(A)**; positive OPLS-DA score plots **(B)**; negative scatter plots of the statistical validations obtained by 200× permutation tests **(C)**; positive scatter plots of the statistical validations obtained by 200 × permutation tests **(D)**; negative volcano plot **(E)**; positive volcano plot **(F)**.

**FIGURE 6:**
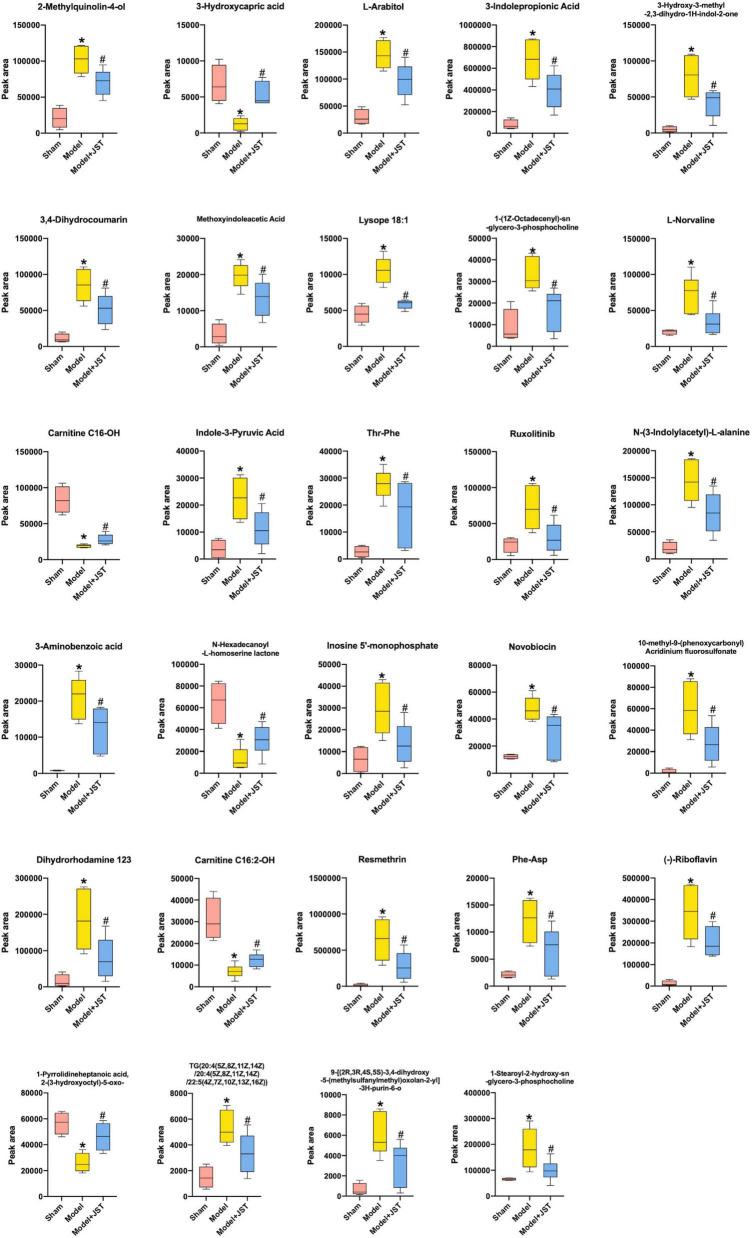
Identified 29 metabolites that were restored by JST treatment in heart failure rats. All data were expressed as mean ± SD (*n* = 4–6). *P < 0.05, vs. Sham group; ^#^*P* < 0.05, vs. Model group.

Orthogonal PLS-DA analysis was applied to maximize the class discrimination and identify potential biomarkers for differentiating metabolites between Sham and Model groups. As shown in Figures 5A,B, the metabolic profiles of Sham and Model groups were completely separated in OPLS-DA score plots. A good explanation and prediction for the OPLS-DA model (Figures 5C,D) was obtained after 200 × permutation tests, with *R*^2^ = 0.9122, *Q*^2^ = −0.661 (negative ion mode) and *R*^2^ = 0.9511, *Q*^2^ = −0.6815 (positive ion mode). The volcano plot visualized and filtered differential metabolites in OPLS-DA mode (Figures 5E,F). A total of 210 metabolites were identified as potential biomarkers between Sham and Model groups based on VIP ≥ 1, *P* < 0.05, and fold change ≥ 2 or fold change ≤ 0.5. Among these biomarkers, 29 metabolites were significantly restored by JST back to Sham level (Figure 6 and Table 1). The identified 29 biomarkers mainly included amino acids [L-norvaline, N-(3-Indolylacetyl)-L-alanine, Phe-Asp, Thr-Phe, etc.], lipids (triglycerides, carnitine, etc.), and sugars (L-arabinitol, etc.).

**TABLE 1:** Identified potential biomarkers regulated by JST.

Mode	Metabolite	Formula	m/z	RT (min)	VIP	Fold change	Trend (Model vs. Sham)	Trend (Model + JST vs. Model)
ESI–	2-Methylquinolin-4-ol	C10H9NO	158.0612	5.3157	1.73982	0.69235	↑^#^	↓*
	3-Hydroxycapric acid	C10H20O3	188.1371	5.0249	1.78929	3.72802	↓^#^	↑*
	L-Arabitol	C5H12O5	204.0663	5.324	1.68952	0.67378	↑^#^	↓*
	(-)-Riboflavin	C17H20N4O6	375.1349	5.324	1.67590	0.45535	↑^#^	↓*
	3-Indolepropionic acid	C11H11NO2	188.0718	5.324	1.64619	0.58819	↑^#^	↓*
	3-Hydroxy-3-methyl-2,3-dihydro-1H-indol-2-one	C9H9NO2	184.0402	5.8226	1.64685	0.52641	↑^#^	↓*
	3,4-Dihydrocoumarin	C9H8O2	189.0752	5.3241	1.59275	0.61151	↑^#^	↓*
	Methoxyindoleacetic acid	C11H11NO3	205.0693	5.324	1.56725	0.68438	↑^#^	↓*
	Lysope 18:1	C23H46NO7P	550.3022	8.4159	1.81812	0.63511	↑^#^	↓*
	1-(1Z-Octadecenyl)-sn-glycero-3-phosphocholine	C26H54NO6P	592.3369	8.9885	1.74370	0.52359	↑^#^	↓*
	L-Norvaline	C5H11NO2	273.1208	0.8031	1.79509	0.45803	↑^#^	↓*
	Carnitine C16-OH	C23H45NO5	416.3367	7.3087	1.82732	1.58737	↓^#^	↑*
	Indole-3-pyruvic acid	C11H9NO3	408.1287	5.3152	1.65767	0.49106	↑^#^	↓*
	1-Pyrrolidineheptanoic acid, 2-(3-hydroxyoctyl)-5-oxo-	C19H35NO4	342.2638	6.08	1.73222	2.10671	↓^#^	↑*
	Ruxolitinib	C17H18N6	391.1283	5.3156	1.64617	0.41975	↑^#^	↓*
	N-(3-Indolylacetyl)-L-alanine	C13H14N2O3	247.1074	5.317	1.62870	0.59240	↑^#^	↓*
	3-Aminobenzoic acid	C7H7NO2	155.0807	1.526	1.58318	0.58909	↑^#^	↓*
	N-Hexadecanoyl-L-homoserine lactone	C20H37NO3	386.2898	6.1953	1.66734	2.32087	↓^#^	↑*
ESI+	Inosine 5’-monophosphate	C10H13N4O8P	425.1337	5.3159	1.58944	0.46321	↑^#^	↓*
	Novobiocin	C31H36N2O11	613.2392	8.0814	1.56260	0.60881	↑^#^	↓*
	10-methyl-9-(phenoxycarbonyl) acridinium fluorosulfonate	C21H15NO2	331.1433	5.3169	1.57713	0.45962	↑^#^	↓*
	Dihydrorhodamine 123	C21H18N2O3	393.1438	5.3161	1.57769	0.42733	↑^#^	↓*
	TG(20:4(5Z,8Z,11Z,14Z)/20:4(5Z,8Z,11Z,14Z)/22:5 (4Z,7Z,10Z,13Z,16Z))	C65H100O6	1054.6896	9.1449	1.48480	0.62950	↑^#^	↓*
	Resmethrin	C22H26O3	377.1492	5.3163	1.55403	0.43515	↑^#^	↓*
	Phe-Asp	C13H16N2O5	281.112	2.5847	1.45288	0.54956	↑^#^	↓*
	1-Stearoyl-2-hydroxy-sn-glycero-3-phosphocholine	C26H54NO7P	568.3373	8.9370	1.54656	0.53681	↑^#^	↓*
	9-[(2R,3R,4S,5S)-3,4-dihydroxy-5-(methylsulfanylmethyl)oxolan-2-yl]-3H-purin-6-one	C11H14N4O4S	342.1073	3.0636	1.46831	0.54309	↑^#^	↓*
	Thr-Phe	C13H18N2O4	267.134	1.1521	1.45634	0.53456	↑^#^	↓*
	Carnitine C16:2-OH	C23H41NO5	394.2949	6.4516	1.64133	2.06973	↓^#^	↑*

^#^*P* < 0.05, vs. Sham group, *P < 0.05, vs. Model group. ↑Represents an increase and ↓represents an decrease for the comparison of Model group vs. Sham group or JST + Model group vs. Model group.

## Discussion

Heart failure is a chronic and progressive condition of various cardiovascular diseases that cause heart damage (23, 24). Heart failure affects more than 64.3 million people worldwide with the rate of hospitalization continuing to rise every year. Heart failure causes peripheral congestion, pulmonary edema, and exercise intolerance, leading to nausea, dyspnea, loss of appetite, reduced exercise capacity, and a poor prognosis for heart failure patients (25). Meanwhile, the annual global expenditure related to the treatment of heart failure is estimated at as much as $108 billion (26–28). In the present study, we first successfully established a rat model of heart failure by LAD ligation, followed by comprehensively assessing therapeutic effects and mechanisms of JST on the treatment of heart failure. The results demonstrated that JST could improve the cardiac function of heart failure rats via enhancing rat LVEF and LVFS and reducing LVIDd, LVIDs, IVSd, and IVSs. JST also reduced the serum LDH activity and the level of NT-pro BNP, markers of heart failure and myocardial damage, and inhibit myocardial fibrosis to delay ventricular remodeling, thus exerting therapeutic effects for the treatment of heart failure.

**FIGURE 7:**
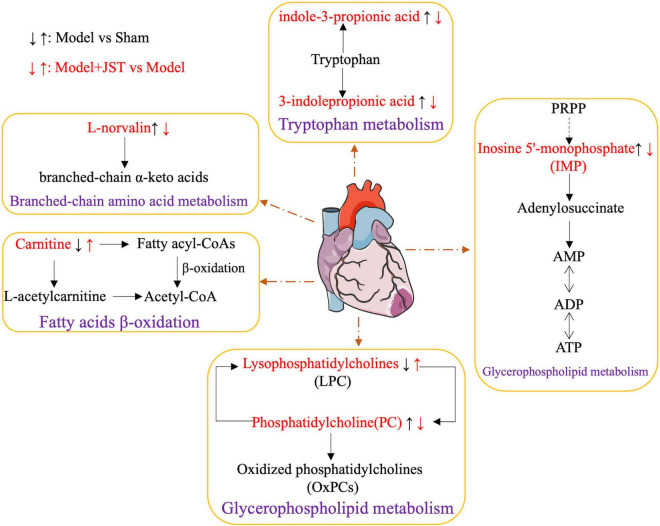
Network of the potential biomarkers associated with heart failure. The metabolites written in red are identified as potential biomarkers. “↑” represents upregulation, “↓” represented downregulation.

Metabolic disorders are closely associated with heart failure because they can lead to structural abnormalities of cardiac myocytes and endothelial dysfunction (29, 30). To further delineate the underlying mechanisms of JST against heart failure, the metabolomics based on UHPLC-Q-TOF-MS/MS method was applied to investigate the changes in the metabolic processes of heart failure and the therapeutic effects of JST. According to the metabolic profiling analysis, Sham, Model, and JST groups were clearly separated in the PCA and PLS-DA scores plots, and OPLS-DA analysis revealed that a total of 29 metabolites that varied significantly between Sham and Model groups were restored by the treatment of JST. Tryptophan metabolism, branched-chain amino acid metabolism, fatty acids β-oxidation, and glycerophospholipid metabolism were closely related to those metabolites, representing the major pathways involved in the therapeutic effects of JST on heart failure (Figure 7).

Tryptophan metabolism is closely linked to various cardiovascular diseases. Accelerated catabolism of tryptophan and thus the resultant increase of serum kynurenine to tryptophan ratios are frequently observed in people with cardiovascular diseases, and increased tryptophan breakdown adds to the detrimental effects of pre-existent reduced vascular reserves (31). Studies on a cell level demonstrated that indole-3-propionic acid, a microbial metabolite of tryptophan, induced the enhancement of maximal mitochondrial respiration with acute treatment, while it led to mitochondrial dysfunction following chronic exposure in cardiomyocytes (32). Currently, the impact of tryptophan and its microbiota-derived metabolites on cardiac functions is still controversial. In the present study, we found that the level of gut microbiota-derived tryptophan metabolites, 3-indolepropionic acid, and indole-3-pyruvic acid, remarkably elevated in model rats, suggesting the potential increase of gut microbiota-derived metabolism of tryptophan and thus the increased formation of corresponding metabolites. The level of these metabolites was significantly reduced by JST treatment, which indicated that the gut microbiota-related tryptophan metabolism pathway might be associated with the therapeutic effects of JST on heart failure.

Catabolic defect of branched-chain amino acids such as L-norvaline contributes to cardiac dysfunctions. This metabolic defect leads to the accumulation of branched-chain α-keto acids, which directly impairs mitochondrial activity, induces oxidative stress, and exacerbates cardiac dysfunction. It was found that restoration of L-norvaline catabolism by drugs can slow the disease progression of pressure overload-induced heart failure (33). In addition, plasma L-norvaline levels were significantly higher in patients with dilated cardiomyopathy and ischemic cardiomyopathy than that in healthy volunteers (34). In the present study, the level of plasma L-norvaline was significantly increased in heart failure rats and reduced significantly by JST treatment, suggesting that JST alleviated heart failure by improving the defective catabolism of branched-chain amino acids, and L-norvaline might serve as a biomarker for the diagnosis and treatment of heart failure.

Lipid metabolism plays a crucial role in maintaining the structural and functional integrity of the heart, and the coordinated control of fatty acid uptake, β-oxidation, and mitochondrial oxidative phosphorylation by cardiac myocytes is essential for ATP production in the heart. Previous studies found that the heart produced ATP mainly through the consumption of fatty acids contributing to 40–60% of ATP production (35, 36). Triglyceride (TG) is a source of essential fatty acids for generating myocardial ATP, and the metabolism of TG is critical in regulating myocardial lipid metabolism. Moreover, excessive accumulation of lipids in the heart can disrupt normal cell-cell interaction, leading to apoptosis, cardiac hypertrophy, and cardiac dysfunction (35, 36). Cardiac pressure overload causes the dysfunction of mitochondrial substrate oxidation and respiration, excessive lipid accumulation, and heart failure (37–39). In the present study, the plasma levels of various lipids, such as TG (20:4(5Z,8Z,11Z,14Z)/20:4(5Z,8Z,11Z,14Z)/22:5(4Z,7Z,10Z,13Z, 16Z)), Lysope18:1, 1-(1Z-octadecenyl)-sn-glycero-3-phospho- choline, inosine 5’-monophosphate, 1-stearoyl-2-hydroxy-sn-glycero-3-phosphocholine, were remarkably higher in Model rats compared to that in Sham rats and were significantly reduced by JST treatment, indicating that JST might restore the lipid metabolism including fatty acids and glycerophospholipid metabolism in heart failure rats.

Carnitine is essential for fatty acid oxidation in cardiac myocytes, which promotes the transfer of long-chain fatty acids across the inner mitochondrial membrane for subsequent β-oxidation. Moreover, L-carnitine may also regulate calcium influx, endothelial integrity, intracellular enzyme release, and membrane phospholipid content for sustained cellular homeostasis and inhibit myocardial fibrosis via the arachidonic acid metabolic pathway (39, 40). Treatment with carnitine resulted in the reduction of ROS production and myocardial infarct size in cardiac myocytes (41–43). In the present study, the levels of carnitine C16-OH and carnitine C16:2-OH were significantly lower in Model rats compared to that in Sham rats, and were enhanced by JST treatment, further confirming the potential therapeutic mechanisms of JST for treating heart failure via regulating fatty acid β-oxidation pathway.

## Conclusion

The present study demonstrated that JST could improve the cardiac function, reduce the serum LDH activity and the level of NT-pro BNP, and inhibit myocardial fibrosis of heart failure rats to exert its therapeutic effects. The untargeted metabolomics analysis revealed that a total of 29 metabolites that altered significantly in heart failure rats were restored by the treatment of JST. These metabolites were mainly involved in tryptophan metabolism, branched-chain amino acid metabolism, fatty acids β-oxidation, and glycerophospholipid metabolism. The present findings provided new insights into the mechanism of JST in the treatment of heart failure via restoring amino acid metabolism and lipid metabolism in heart failure rats.

## Data availability statement

The raw data supporting the conclusions of this article will be made available by the authors, without undue reservation.

## Ethics statement

This animal study was reviewed and approved by the Committee on Laboratory Animal Care and Use of Guangdong Pharmaceutical University.

## Author contributions

XH, JM, and YH contributed to the design concepts of this whole study. XM and JC carried out the animal experiment, performed data analyses, and drafted the manuscript. YS and JL carried out the animal experiment and collected data. All authors have read and approved the content of the manuscript.

## Footnotes

1
https://proteowizard.sourceforge.io/tools/msconvert.html

2
http://www.bioconductor.org/packages/release/bioc/html/xcms.html

3
http://www.massbank.jp

4
http://www.hmdb.ca/

5
https://metlin.scripps.edu
