# Supplementary material for: Plasma metabolomic analysis reveals the therapeutic effects of Jiashen tablets on heart failure
Source: Front Cardiovasc Med. 2022 Dec 6;9:1047322. doi: 10.3389/fcvm.2022.1047322 (PMC9763324; doi:10.3389/fcvm.2022.1047322)
Supplement: Supplementary file 1 [file Data_Sheet_1.pdf]

# *Supplementary Material*

## 1 Supplementary Note

### 1.1 The procedures for the preparation of JST

Huangqi (Astragali Radix), Danshen (Salviae Miltiorrhizae Radix et Rhizoma), Xiangjiapi (Periplocae Cortex), Sanqi (Notoginseng Radix et Rhizoma), Yimucao (Leonuri Herba), Chenpi (Citri Reticulatae Pericarpium), Guizhi (Cinnamomi Ramulus), and Tinglizi (Descurainiae Semen Lepidii Semen) were used for the preparation of JST. Danshen, Xiangjiapi, Huangqi, and Sanqi were extracted with dilute ethanol solution and were separated and concentrated by macroporous adsorption resin to generate the extracts. Guizhi, Yimucao, Tinglizi, and Chenpi were extracted with water and then precipitated by ethanol solution to produce corresponding extracts. The above-mentioned extracts were proportionally mixed to give JST.

## 2 Supplementary Figures

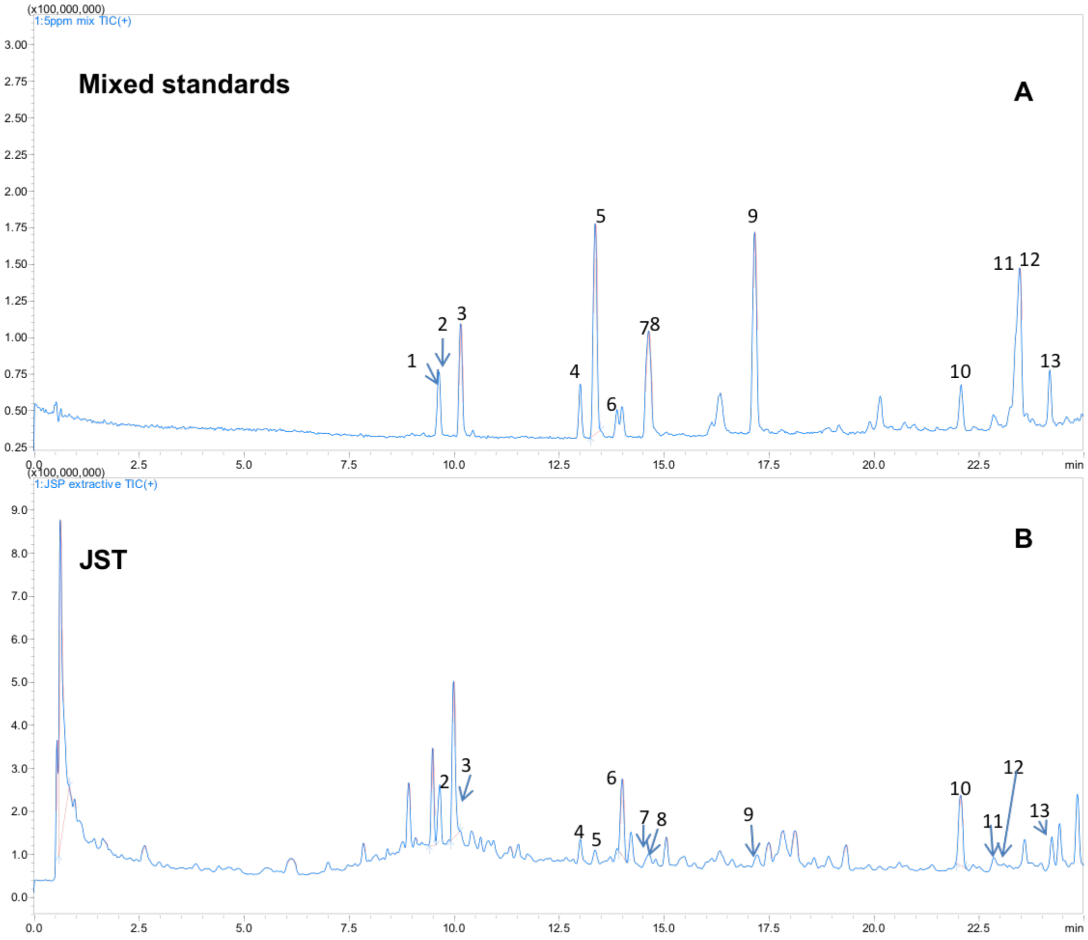


**Figure S1.** Total ion chromatogram (TIC) for mass spectrometric analysis of mixed standards (A) and JST samples (B). The analysis was conducted on a Shimadzu LC-40D triple quadrupole (QQQ) mass spectrometry connecting with an Shimadzu LCMS-8045 ultra-high performance liquid chromatography (UPLC) (Shimadzu Co.,Ltd, Japan). Chromatographic separation was achieved on ACQUITY BEH C18 column (2.1mm×100mm, 1.7 μ m) at 45℃ with mobile phase of 0.1% formic acid (A) and acetonitrile (B). The elution gradient was as follows: 0-3 min, 20% A; 3-9 min, 20~30% A; 9-17 min, 30~50% A; 17-20 min, 50~90% A; 20~25 min, 90% A. The flow rate was 0.40 mL/min and the injection volume was 1 μL. 1, 4-methoxy-salicylic acid; 2, rosmarinic acid; 3, ononin; 4, notoginsenoside R1; 5, periplogenin; 6, ginsenoside Re; 7, ginsenoside Rg1; 8, formononetin; 9, periplocymarin; 10, ginsenoside Rb1; 11, astragaloside IV; 12, periplocoside M; 13, ginsenoside Rd.

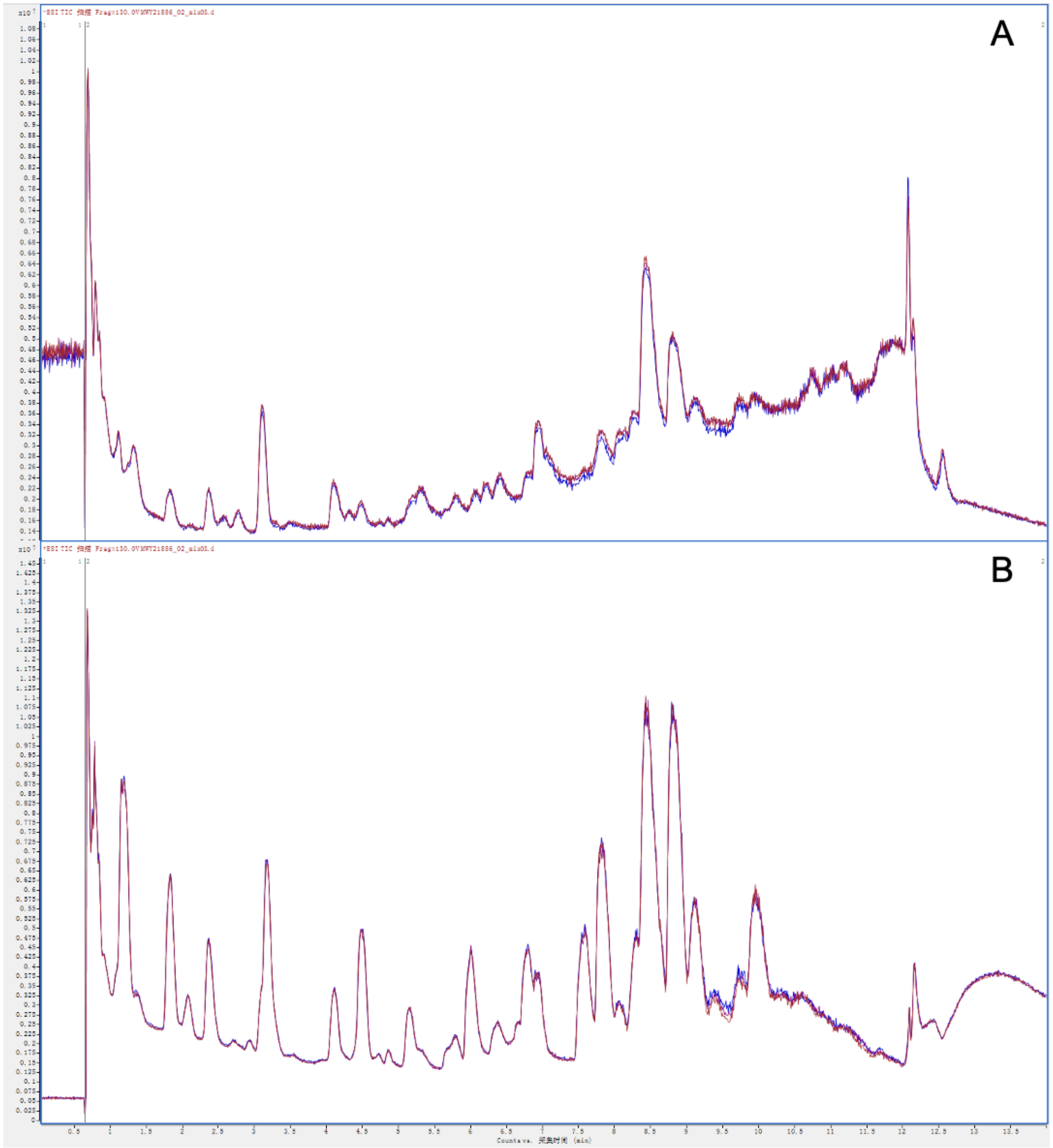


**Figure S2.** Total ion chromatogram (TIC) overlap map for mass spectrometric analysis of QC samples under positive ion mode (A) and negative ion mode (B). TIC: the spectrum obtained by adding the intensities of all ions in the mass spectrometry at each time point is continuously depicted; the abscissa is the retention time (RT) for metabolite detection, and the ordinate is the ion current intensity (intensity unit is cps, count per second) for ion detection.

## 3 Supplementary Table

**Table S1.** The content of major constituents in JST

| Compounds in JST | content（mg/g） |
|---|---|
| 4-Methoxysalicylic acid | 0.117464 |
| Ononin | 0.119512 |
| Astragaloside IV | 0.373903 |
| Periplocymarin | 0.019913 |
| Ginsenoside Rg1 | 0.566996 |
| Ginsenoside Rd | 0.141482 |
| Ginsenoside Re | 0.567285 |
| Notoginsenoside R1 | 0.672153 |
| Ginsenoside Rb1 | 2.441462 |
| Periplogenin | 0.018853 |
| Formononetin | 0.037781 |
